# The use of selected electrophoretic techniques to assess the health of domestic cats *(Felis catus)*

**DOI:** 10.2478/jvetres-2025-0062

**Published:** 2025-10-30

**Authors:** Bartosz Jania, Katarzyna Andraszek, Ewa Wójcik, Maria Dmitruk

**Affiliations:** Institute of Animal Science and Fisheries, University of Siedlce, 08-110 Siedlce, Poland

**Keywords:** cat, electrophoresis, comet assay, infectious diseases, diagnostics

## Abstract

**Introduction:**

Electrophoretic analytical techniques provide extremely important information about an animal’s clinical condition. They are recommended in every case, including in screening tests of animals showing no concerning clinical symptoms. Such tests can detect subclinical conditions, such as inflammation, antigen stimulation or certain forms of cancer. The aim of the study was to determine the suitability of native serum protein electrophoresis and the comet assay for assessing the health status of cats.

**Material and Methods:**

Electrophoresis was performed on serum samples from 125 cats. On sera with abnormalities in electropherograms (25 individuals), the following additional analyses were performed: haematological analysis, microscopic examination of a blood smear, plate tests detecting antibodies against feline infectious peritonitis (FIP) and feline immunodeficiency virus (FIV), a plate test detecting feline leukaemia virus (FeLV) surface antigen and a comet assay in peripheral blood lymphocytes.

**Results:**

Native protein electrophoresis enabled the identification of latent disease conditions in individuals assessed as good for overall condition on the basis of clinical examination. Some cats thus assessed had an abnormal electropherogram and were carriers of FIV, FeLV or FIP. In addition, the comet assay identified increased instability in the genetic material of cats with electropherogram abnormalities.

**Conclusion:**

Electrophoretic techniques can be successfully used as a tools for identifying latent conditions and evaluating the overall health status of cats.

## Introduction

Serum protein electrophoresis provides valuable information on an animal’s health, constituting a basis for assessment of its overall condition. Even in the absence of visible clinical symptoms (*i.e*. in subclinical forms of a disease), the analysis reveals conditions such as inflammation, increased antigen stimulation, certain forms of cancer, immune deficiencies or malnutrition. The basic indication for serum protein electrophoresis is a high or low total protein level in the serum. Even when the protein concentration is within the reference range, serum protein electrophoresis should be included in the recommended basic panel of laboratory tests for cats, not only to diagnose the causes underlying clinical symptoms, but also as one of several screening tests for assessment of the animal’s overall condition. Another method using electrophoretic separation is the comet assay, which analyses DNA strand breaks in individual cells. This method identifies damage to single and double DNA strands and chromatids. This type of damage is induced by genotoxic factors (*e.g*. free radicals, ionising radiation or compounds coming into contact with nucleic acids). The analysis can be conducted on any cells that can be maintained in a suspension.

Proteins play a role in maintaining homeostasis in the body. Disturbances of homeostasis lead to disease and even death. Therefore, normal concentrations of individual serum protein fractions must be maintained. Proteins are the most important structural and functional group of chemical compounds, responsible for the structures comprising living organisms and the processes taking place in them. Some proteins are present in blood serum in amounts measured in g/mL (such as albumins, immunoglobulins, haptoglobin, transferrin and lipoproteins) (1), while others (such as hormones) are measured in ng/mL or pg/mL. The concentrations of individual proteins are strictly controlled in the body in order to maintain their functions in areas such as immunity, blood clotting, small molecule transport and control of inflammation. An imbalance in the serum concentrations of individual proteins or dysfunctions of these proteins can be a cause or an effect of an ongoing disease process (18).

The method of electrophoretic separation of proteins in body fluids has been known for more than one hundred years (7). In diagnosis of human diseases, characteristic electropherograms are ascribed to numerous conditions, including inflammatory states and malignant tumours (6). Native protein electrophoresis has been used as a diagnostic technique in human medicine for more than 40 years (4). It is only in the last 20 years, however, that electrophoretic techniques have begun to be used in veterinary medicine (26). Electrophoresis is rarely the main diagnostic tool, but it is used in clinical cases involving acute and chronic inflammation or immune stimulation. In cats, it can be a supporting technique in the diagnosis of feline infectious peritonitis (FIP), feline leukaemia (caused by the feline leukaemia virus – FeLV) or feline immunodeficiency syndrome (caused by the feline immunodeficiency virus – FIV) (4, 25).

Following electrophoretic separation, individual protein fractions and subfractions are stained and are quantified on the basis of the intensity of the colour of the corresponding bands. This is currently done by computer software; the system calculates the content of the protein on the basis of the optical density of individual bands (6). During separation, the original mixture of proteins is usually divided into five basic fractions: albumins, α_1_-globulins, α_2_-globulins, β-globulins and γ-globulins (4). In some cases, the beta fractions are divided into β_1_ and β_2_, and sometimes the gamma fraction is divided into γ_1_ and γ_2_ (13, 26). Each globulin fraction consists of acute phase proteins or antibodies, and sometimes both (4).

The alpha globulin fraction contains proteins such as α_1_-fetoprotein, α_1_-acid glycoprotein, α_1_-antitrypsin (a protease inhibitor), α_1_-antichymotrypsin (a protease inhibitor), α_1_-lipoprotein (high-density lipoprotein, a lipid transporter), ceruloplasmin (a copper transporter), haptoglobin (which binds haemoglobin), α_2_-macroglobulin (a protease inhibitor) and serum amyloid A (23, 26). The beta globulin fraction includes β_2_-lipoprotein (low-density lipoprotein, a fat transporter), transferrin (an iron transporter), ferritin (which stores iron), complement system components (C3 and C4) and fibrinogen (in plasma, but not in serum) (5, 19, 23, 26). Class M and A immunoglobulins may migrate into the β region as well. Free haemoglobin also migrates into this fraction if it is present in the sample (*e.g*. in the case of intravascular haemolysis or *in vitro*, when haemoglobin is released into the serum before being separated from the blood cells) (13). The γ-globulin fraction is highly varied and includes various classes of immunoglobulins. Antibodies are produced by plasma cells in response to antigen stimulation. In dogs, C-reactive protein also migrates in this fraction (30). In horses, it is found in the region between β and γ (24).

An electrophoretic technique widely used to evaluate the stability of genetic material is the comet assay (single-cell gel electrophoresis) (21). This is a simple method for measuring damage to DNA by detecting strand breaks in eukaryotic cells. Cells embedded in agarose on a microscope slide are lysed with a detergent in high salinity in order to separate the strands of nucleoids containing supercoiled loops of DNA linked to the nuclear matrix. Electrophoresis in high pH conditions and staining with ethidium bromide reveals structures reminiscent of a comet, which can be examined under a fluorescence microscope (2, 16, 21). The ratio of the intensity of the fluorescence emission of the comet tail to that of the head is proportional to the number of DNA breaks. The basis for this phenomenon is most likely the loss by loops containing DNA strand breaks of their superhelical structure and consequent freedom to stretch towards the anode (21). This assay is used to test new chemical substances for genotoxicity, as well as for monitoring contamination of the environment with genotoxins, in epidemiological research and in basic research on the mechanisms of DNA damage and repair (27, 29). The sensitivity and specificity of the assay are much higher if the nucleoids are incubated with bacterial repair endonucleases, which recognise specific types of DNA damage and convert the damage to DNA breaks, increasing the amount of DNA in the tail of the comet. Repair of DNA can be monitored if cells are incubated with a damaging agent and the damage is measured at time intervals. Alternatively, repair activity in a cell extract can be measured by incubating it with nucleoids containing specific damage. The analysis can be performed on various types of tissues comprising cells which can be converted into a suspension (2, 16, 21).

Slides are analysed under a fluorescence microscope using the particular filter for the stain used. The available equipment determines how results are read, but primarily they are by visually determination of the degree of DNA fragmentation (the amount of DNA in the tail of the comet) and classification of what is seen to one of four categories. If a camera and image analysis software can be used, it is possible to obtain more precise data. Instability of genetic material detected in this way is a reflection of population or individual sensitivity to genotoxic environmental factors. It can be used to assess the risk of occupational diseases (10, 11), to evaluate genome integrityand as a cytogenetic marker in the biomonitoring of selected populations (12, 27–29).

The aim of this study was to determine the suitability of selected electrophoretic techniques for potential inclusion in a screening test panel for evaluating the overall health of cats.

## Material and Methods

### Material

The material for the study consisted of the blood of 125 domestic cats of unknown age and sex from Siedlce and its vicinity. The cats had been brought to a veterinary clinic under the ‘Programme for the care of homeless animals and prevention of animal homelessness in the city of Siedlce’, under an agreement between the clinic and the city of Siedlce. The overall condition of all cats was assessed, and in most cases it was good. No imaging was performed. Cats were selected for detailed analyses on the basis of native serum protein electrophoresis. In cats with abnormal electropherograms (25 individuals), the following additional analyses were performed: haematological analysis, microscopic examination of a blood smear, plate tests detecting antibodies against FIP and FIV, a plate test detecting FeLV surface antigen and a comet assay in peripheral blood lymphocytes. In addition, the total protein concentration in the serum was determined, and the albumin to globulin ratio was calculated.

### Blood collection

Blood was drawn from the external jugular vein or cephalic vein of each cat during routine veterinary procedures. A 1 mL sample was placed in a tube containing ethylenediaminetetraacetic acid as an anticoagulant for subsequent analysis. This sample was examined in a haematology analyser, a smear was prepared from it for staining and microscopic evaluation, and the comet assay was run on it. A 2 mL sample was placed in a tube without anticoagulant to obtain serum for immunochromatographic analysis and native protein electrophoresis. The sample collected into the tube with anticoagulant was analysed for white blood cell count (WBC), red blood cell count (RBC), haemoglobin (HGB), haematocrit (HCT), platelet count, mean corpuscular volume (MCV), mean corpuscular haemoglobin concentration (MCHC), lymphocyte percentage, monocyte percentage, granulocyte percentage, lymphocyte count (LYM), monocyte count (MON) and granulocyte count (GRA).

### Microscopic evaluation of blood smears

Stained and dried slides were analysed under an Olympus BX 50 light microscope (Olympus, Tokyo, Japan) fitted with a ProgRes digital camera (Jenoptik, Jena, Germany) at 1000× magnification (using an immersion lens with 100× magnification and a 10× eyepiece), applying a standard microscope procedure for evaluating a stained peripheral blood smear. The smears were searched in particular for features indicating ongoing inflammation (increased total leukocyte count and abnormalities in the proportions of leukocyte types).

### Immunoassays

The presence of antibodies against FIV, or of feline leukaemia virus (FeLV) antigen was tested using the qualitative Rapid FIV Ab/FeLV Ag immunoassay (VetExpert, Warsaw, Poland). The assay is based on the chromatographic method and uses filter paper with inactivated antigen or antibody (depending on whether antibody or antigen is being detected).

### Native serum protein electrophoresis

Serum was obtained from blood samples collected into tubes without anticoagulant and analysed by native protein electrophoresis using the SAS-MX Serum Protein SB kit (Helena Biosciences, Gateshead, UK). From 4 to 6 fractions (albumins, α- and β-globulins, and γ-globulins) were distinguished on the electropherogram, and their proportions were given. Absolute values were calculated by substituting the total protein concentration obtained using a refractometer.

### Comet assay

Twenty-five cats for which native serum protein electrophoresis revealed clear abnormalities were selected for the comet assay. In addition, a comparative analysis was performed using the assay on 25 cats without abnormalities in their electropherograms. These cats were clinically assessed as healthy. The assay was performed according to the single-cell gel electrophoresis (SCGE) procedure (21). Lymphocytes centrifuged from whole blood were suspended on microscope slides covered with a layer of 0.5% normal melting point agarose gel, and then embedded twice in 0.5% low melting point agarose gel. Alkaline lysis was used to release DNA from the cell and remove proteins. The next step was alkaline denaturation in an electrophoresis solution, which was followed by electrophoresis (25 V, 300 mA, 20 min), neutralisation with Tris-HCl and staining with ethidium bromide. A total of 50 cells (comets) from each individual were examined under the Olympus BX 50 light microscope with a digital camera. Cells were analysed and archived at 40× magnification. Cells with overlapping images or located close to the edge of the slide were not analysed. The head and tail of the comet were placed in the measurement frames. Assessment of the DNA integrity of the cells was based on the percentage content of nuclear DNA in the head (Head DNA%) and tail (Tail DNA%) of the comet. Individuals with Head DNA% above 90 were considered to have normal DNA integrity, while with less than 90 were classified as having impaired DNA integrity. The CASP tool, v. 1.2.0 was used for image analysis (3).

Damage (determined by the comet assay) in individual cats was compared by one-way analysis of variance according to the following mathematical model:

$${\text{y}}_{\text{ij}}\;=\;\text{m}\;+\;{\text{a}}_{\text{i}}\;+\;{\text{e}}_{\text{ij}}$$

where y_ij_ is the value of the trait (% damage shown on the basis of Head DNA% and Tail DNA%), m is the grand mean, ai is the effect of the i^th^ level of a factor (healthy or sick individual), and e_ii_ is the sampling error.

Detailed comparison of means (*post-hoc* analysis) was carried out using Tukey’s test at P-value ≤ 0.05.

The degree of chromosome damage was compared between sick and healthy individuals (evaluated on the basis of Head DNA% and Tail DNA%) using Student’s *t*-test for independent samples with equal variances, according to the following formula:

$$t\;=\;\backslash frac\left\{\bar{x_{1}-{\bar{x}}_{2}}\right\}\left\{\sqrt{\left\{\backslash frac\left\{s_{1}^{2}\right\}\left\{n_{1}\right\}+\backslash frac\left\{s_{2}^{2}\right\}\left\{n_{s}\right\}\right\}}\right\}$$

where

${\bar{x}}_{1};\;{\bar{x}}_{2}$ the means from samples, n_1_; n_2_ are the sample size, and $S_{X_{1}}^{2};\;S_{x_{2}}^{2}$ is the sample variance.

Equality of variance was tested by an F-test as follows:

$$F_{0}=\frac{\frac{{}^{n}s_{x_{1}}^{2}}{n_{1}-1}}{\frac{{}^{n}s_{x_{2}}^{2}}{n_{2}-1}}$$

Significance was tested for a P-value ≤ 0.05.

Statistical analysis of the results of the comet assay was performed using the Statistica data analysis software system, v. 13 (Dell, Round Rock, TX, USA).

## Results

**Fig. 1.**
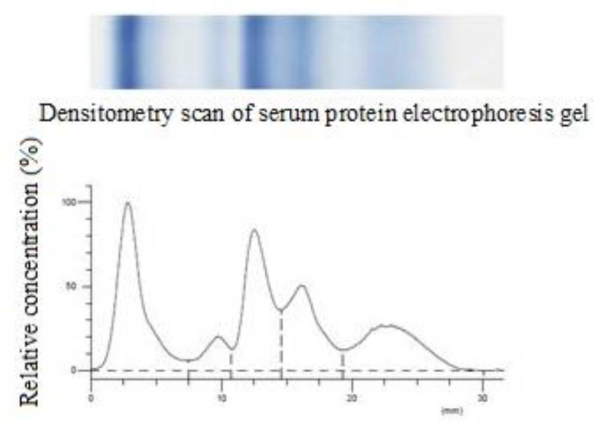
Electrophoretic separation and histogram – cat No. 1

Each cat in which abnormalities were found in electrophoresis was characterised in terms of the results of the analyses described above. As an example, a description of one case (cat No. 1) and the results of native serum protein electrophoresis correlated with immunoassays and haematological tests are detailed below. The overall condition of the cat was good, and it had negative test results for FIV and FIP, although the FeLV test result was positive. The results of the analysis of blood samples using a haematology analyser, the concentrations of individual protein fractions, and the results of the comet assay are presented in Tables 1, 2 and 3. The electrophoretic separation is shown in Fig. 1.

**Table 1.** Haematological analysis – cat No. 1

Parameter	Result	Reference
WBC	4.90↓	6.0–18.0×10^3^/mm^3^
RBC	3.31↓	6.5–10.0×10^6^/mm^3^
HGB	3.40↓	6.2–9.3 mmol/L
HCT	0.15↓	0.30–0.45
MCV	46	39–55 μm^3^
MCHC	22.3	18.6–22.3 mmol/L
PLT	230	200–600×10^3^/mm^3^
%LYM	50	20–55 %
%MON	2	1–10 %
%GRA	48	35–75 %
LYM	2.5	1.2–3.2×103/mm^3^
MON	0.1↓	0.3–0.8×103/mm^3^
GRA	2.4	1.2–6.8×10^3^/mm^3^

1 WBC – white blood cells; RBC – red blood cells; HGB – haemoglobin; HCT – haematocrit; MCV – mean corpuscular volume; MCHC – mean corpuscular haemoglobin concentration; PLT – platelets; %LYM – lymphocyte percentage; %MON – monocyte percentage; %GRA – granulocyte percentage; LYM – lymphocytes; MON – monocytes; GRA – granulocytes

**Table 2.** Concentrations of individual protein fractions in serum protein electrophoresis – cat No. 1

Fraction	Relative area (%)	Concentration (g/L)	Range (g/L)	Albumin-to-globulin ratio
Albumins	29.93	20.06↓	27–39	
α-globulins	30.96	20.74↑	6.5–12.1	
β-globulins	19.72	13.21↑	4.5–8.7	
γ-globulins	19.39	12.99	10.0–18.6	
Total protein		67	60–80	
A/G ratio				0.43

**Table 3.** Comet assay results on lysed whole blood – cat No. 1

Head DNA (%)		Tail DNA (%)	
Mean	Standard deviation	Mean	Standard deviation
64.21	29.10	35.79	29.10

**Fig. 2.**
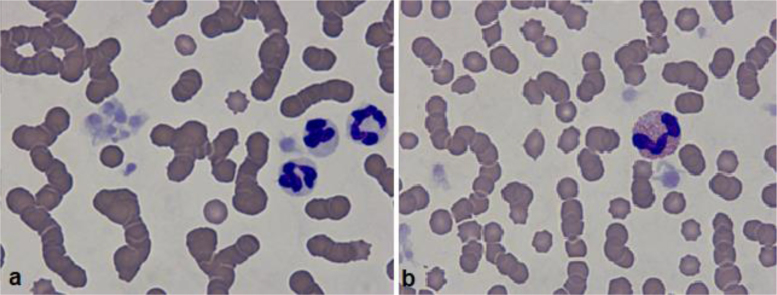
Cytology slide – cat No. 1. (a) neutrophil granulocytes, platelets and red blood cells, visible rouleaux; (b) eosinophilic segmental granulocyte, platelets and red blood cells, visible rouleaux (b)

In the haematological analysis of this cat, the low total WBC count is notable. However, the various leukocyte subgroup parameters were within reference ranges (apart from the slightly low MON count). Nevertheless, the erythrocyte parameters (RBC, HGB and HCT) were lower, which indicated anaemia. Giving consideration to the MCV and MCHC being within reference ranges, it did not appear to be anaemia associated with iron deficiencies. Protein electrophoresis revealed a low concentration of the albumin fraction. This is usually due to the production of other proteins, such as acute-phase proteins, or to anaemia or blood loss. Markedly elevated levels of α- and β globulins could also be seen. This indicated an increased concentration of acute-phase proteins, which pointed to ongoing inflammation. The microscope photograph (Fig. 2) shows neutrophil granulocytes, platelets and red blood cells with characteristic rouleaux. This phenomenon is characteristic for the species, but if it clearly deviates from the norm, it indicates inflammation – increased levels of acute-phase proteins and/or antibodies. The plate tests revealed FeLV infection. Table 3 presents the results of the comet assay. It is important to note the low percentage of DNA remaining in the ‘head’ of the comet: 64.21% of the nucleic acid. This means a low quantity of stable nucleic acid remaining where the lymphocyte was embedded in the agarose gel. In individuals with no pathological changes in the clinical presentation or in *in vitro* analyses, this value should not fall below 90%.

The dominant protein fraction in the serum of healthy cats is albumin. Their level in serum may be elevated in cases of dehydration (*e.g*. cat No. 3), while a reduced concentration may indicate increased globulin production. An elevated concentration was observed in one cat, which had high concentrations of all protein fractions, an increased WBC count and a high total protein concentration. The plate tests for FeLV, FIV and FIP were negative, and the clinical condition of the cat was assessed as good. A reduced albumin concentration was noted in nine cats, but no pattern was observed. The overall health of some of those nine was assessed as good, while some had upper respiratory infections or diarrhoea.

The next important fractions that were clearly distinguishable in the electropherogram were α- and β-globulins. Elevated levels of these proteins may indicate ongoing inflammation. An elevated concentration of at least one of these fractions was detected in 21 cats. In this case, the overall condition of most of them was assessed by a veterinarian as good.

One of the most interesting protein fractions in terms of diagnostics is γ-globulins. This group mainly consists of antibodies. An elevated level of these proteins indicates infection or neoplastic transformation. Sometimes, however, it may be the result of defective proliferation of antibody-producing cells, *i.e*. plasma cells, which are derivatives of B lymphocytes. An increased concentration of gamma globulins was observed in 18 cats, although the overall condition of only some of them was not assessed as good.

An elevated total protein level may be due to dehydration. However, it will also occur in the case of overproduction of one or more globulin fractions. A concentration above 90 g/L indicates intensive production of antibodies belonging to the γ-globulin fraction. A high value for this parameter was noted in eight cats.

Elevated WBC counts, which indicate ongoing inflammation in the body, were also salient observations. A high WBC count was detected in six cats. At the same time, determination of the number of cells in individual white blood cell subpopulations (*e.g*. lymphocytes, granulocytes and monocytes) sometimes enables a closer interpretation. An increased GRA count additionally confirms ongoing inflammation, and an elevated LYM count may indicate conditions such as a viral infection. An elevated WBC count was found in six cats, and a reduced count in four cats. The latter may be significant if the WBC count is considerably below reference values. A very low WBC count was found in cat No. 5, which also had an upper respiratory infection and increased concentrations of α-, β- and γ-globulins.

The results of the plate tests for viral infections were as follows: FIV antibodies were detected in two cats, FeLV antigen was detected in two cats and FIP antibodies were also detected in two cats.

The comet assay identified cats with various degrees of DNA damage. Tail DNA% ranged from 35.79 in cat no. 1 to 16.03 in cat No. 4.

The results of the most important tests are presented together in Table 4.

**Table 4.** Results of protein electrophoresis, immunoassays, haematological analysis and the comet assay in cats with pathological results

	Electrophoresis (g/L)					FIV	FeLV	FIP	Blood count				Comet assay		Overall condition
	Alb	α	β	γ	B. c.				WBC	LYM	MON	GRA	Head DNA%	Tail DNA%	
1	20.06↓	20.74↑	13.21↑	12.99	67	−	+	−	4.90↓	2.5	0.1↓	2.4	64.21	35.79	Good
2	33.91	14.05↑	10.22↑	20.82↑	79	−	−	+	20.40↑	4.7↑	0.2↓	15.5↑	78.9	21.10	Good
3	41.96↑	15.98↑	13.21↑	30.85↑	102↑	−	−	−	21.9↑	8.5↑	2.0↑	11.4↑	83.22	16.78	Good
4	38.76	12.90↑	6.72	21.62↑	80	−	−	−	14.7	4.0↑	1.2↑	9.6↑	83.97	16.03	Good
5	37.59	19.97↑	10.67↑	21.77↑	90↑	+	−	−	2.70↓	0.2↓	0.0↓	2.5	79.95	20.09	Upper respiratory infection
6	33.75	13.66↑	10.45↑	20.14↑	78	−	−	−	9.90	6.2↑	0.3	3.5	80.37	19.63	Good
7	37.49	13.38↑	9.33↑	18.8↑	79	−	−	−	19.8↑	4.0↑	1.2↑	14.6↑	83.11	16.89	Good
8	24.45↓	15.77↑	7.12	25.67↑	73	−	−	−	9.0	3.8↑	0.9↑	4.3	70.47	29.53	Good
9	26.94↓	16.51↑	11.57↑	28.98↑	84↑	−	−	−	13.5	3.9↑	1.2↑	8.4↑	78.38	21.62	Good
10	31.5	12.1	8.19	24.21 ↑	76	−	−	−	5.60↓	1.3	0.2↓	4.0	83.8	16.20	Stomatitis
11	28.00	12.46↑	8.54	32.02↑	81↑	−	−	+	5.60↓	2.0	0.5	3.1	68.08	31.92	Good
12	33.15	12.79↑	10.63↑	25.43↑	82↑	−	−	−	30.8↑	8.3↑	1.5↑	20.9↑	78.54	21.46	Good
13	28.12	10.11	9.05↑	38.72↑	86↑	−	−	−	21.6↑	4.8↑	1.5↑	15.3↑	78.93	21.07	Upper respiratory infection
14	34.52	13.25↑	9.61 ↑	24.62↑	82↑	−	−	−	18.9↑	7.0↑	1.7↑	10.2↑	79.43	20.57	Good
15	31.41	13.52↑	9.84↑	23.23↑	78	−	−	−	12.6	3.8↑	1.3↑	7.6↑	79.92	20.08	Good
16	33.10	16.18↑	11.69↑	9.03↓	70	−	−	−	9.1	3.5↑	0.6	5.0	80.45	19.55	Good
17	32.6	11.72	10.58↑	23.1↑	78	−	−	−	8.9	1.6	0.5	6.8	82.47	17.53	Good
18	27.02	12.58↑	7.19	49.21 ↑	96↑	−	−	−	19.2	6.7↑	1.3↑	11.1↑	78.38	21.62	Upper respiratory infection
19	35.25	8.25	7.66	20.85↑	72	−	−	−	8.5	2.1	0.8	5.6	80.7	19.30	Good
20	24.78↓	13.55↑	10.34↑	12.33	61	−	−	−	7.1	2.0	0.6	4.5	82.78	17.22	Good
21	24.53↓	10.71	6.84	17.93	70	−	+	−	17.7	2.7	1.2↑	13.8↑	65.07	34.93	Good
22	26.12↓	12.65↑	10.55↑	26.68↑	76	−	−	−	6.7	2.0	0.5	4.2	72.36	27.64	Good
23	18.20↓	11.64	5.96	18.20	54↓	+	−	−	15.30	4.0↑	0.8	10.6↑	64.21	35.79	Good
24	25.79↓	15.69↑	8.23	12.29	62	−	−	−	6.70	2.0	0.5	4.2	67.23	32.77	Upper respiratory infection
25	26.05↓	14.42↑	9.57↑	20.96↑	71	−	−	−	8.50	2.9	0.2↓	5.4	67.00	33.00	Upper respiratory infection

### Results of comet assay analyses

**Fig. 3.**
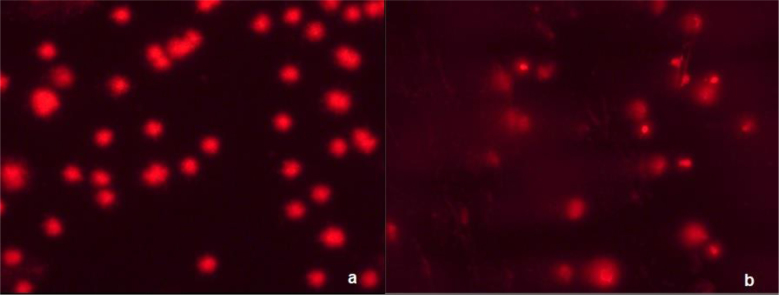
Image of a comet assay on lysed whole blood from a cat. (a) nearly all the genetic material remained in the head of the comet; (b) nearly all the genetic material has migrated to the tail of the comet

The comet assay made it possible to assess the stability of the cats’ genetic material and to distinguish individuals in this regard. The higher the Head DNA% value, the greater the stability of the genetic material and the greater the proportion of DNA located in the head of the comet, *i.e*. in the nucleus of the lymphocyte. This is visible on the microscope slide as an intensely fluorescent circle (Fig. 3a). In the case of disturbed DNA integrity, some DNA is located outside the cell nucleus, in the tail of the comet (Tail DNA% parameter). On the microscope slide, the cell has the shape of a comet, with the damaged DNA in its tail (Fig. 3b). The lower the Head DNA% and the higher the Tail DNA %, the greater the damage to the cell’s DNA.

Detailed results of the comet assay carried out by SCGE are presented in Tables 5 and 6. The average percentage of DNA in the head of the comet in the population was 88.05 ± 11.29, and the percentage in the tail of the comet was 11.95 ± 11.29. The corresponding means for cats with normal DNA integrity were 99.63 ±1.14 (Head DNA%) and 0.37 ± 1.14 (Tail DNA%), while the means for cats with impaired DNA integrity were 76.48 ± 21.45 and 23.52 ± 21.45.

**Table 5.** Means for Head DNA% and Tail DNA% in comet assays on lysed whole blood of cats with normal DNA integrity

Cat	Head DNA%		Tail DNA%	
	Mean	Standard deviation	Mean	Standard deviation
1	99.44^ba^	1.56	0.56^ba^	1.56
2	99.92^a^	0.19	0.08^b^	0.19
3	99.29^ba^	2.22	0.71^ba^	2.22
4	98.29^b^	4.17	1.71^a^	4.17
5	99.59^ba^	0.72	0.41^ba^	0.72
6	99.70^a^	0.61	0.30^b^	0.61
7	99.65^a^	0.64	0.35^b^	0.64
8	99.24^ba^	2.35	0.76^ba^	2.35
9	99.93^a^	0.25	0.07^b^	0.25
10	99.91^a^	0.18	0.09^b^	0.18
11	99.76^a^	0.45	0.24^b^	0.45
12	99.79^a^	0.30	0.21^b^	0.30
13	99.95^a^	0.16	0.05^b^	0.16
14	99.61^a^	1.96	0.39^b^	1.96
15	99.98^a^	0.09	0.02^b^	0.09
16	99.94^a^	0.16	0.06^b^	0.16
17	99.75^a^	1.09	0.25^b^	1.09
18	98.95^ba^	5.78	1.05^ba^	5.78
19	98.91^ba^	3.18	1.09^ba^	3.18
20	99.43^ba^	1.74	0.57^ba^	1.74
21	99.93^a^	0.12	0.07^b^	0.12
22	99.96^a^	0.13	0.04^b^	0.13
23	99.96^a^	0.14	0.04^b^	0.14
24	99.98^a^	0.08	0.02^b^	0.08
25	99.83^a^	0.28	0.17^b^	0.28
Total	99.63	1.14	0.37	1.14

ab – means with different letters are significantly different at P-value ≤ 0.05

**Table 6.** Means for Head DNA% and Tail DNA% in comet assays on lysed whole blood of cats with impaired DNA integrity

Cat	Head DNA%		Tail DNA%	
	Mean	Standard deviation	Mean	Standard deviation
1	64.21^b^	29.10	35.79^a^	29.10
2	78.90^ba^	21.45	21.10^ba^	21.45
3	83.22^a^	14.34	16.78^b^	14.34
4	83.97^a^	17.57	16.03^b^	17.57
5	79.95^ba^	17.56	20.09^ba^	17.56
6	80.37^ba^	18.87	19.63^ba^	18.87
7	83.11^a^	15.90	16.89^b^	15.90
8	70.47^ba^	25.54	29.53^ba^	25.54
9	78.38^ba^	17.23	21.62^ba^	17.23
10	83.80^a^	15.41	16.20^b^	15.41
11	68.08^ba^	32.72	31.92^ba^	32.72
12	78.54^ba^	17.39	21.46^ba^	17.39
13	78.93^ba^	17.47	21.07^ba^	17.47
14	79.43^ba^	17.59	20.57^ba^	17.59
15	79.92^ba^	17.54	20.08^ba^	17.54
16	80.45^ba^	17.56	19.55^ba^	17.56
17	82.47^a^	18.25	17.53^b^	18.25
18	78.38^ba^	17.23	21.62^ba^	17.23
19	80.70^ba^	17.79	19.30^ba^	17.79
20	82.78^a^	18.44	17.22^b^	18.44
21	65.07^b^	29.93	34.93^a^	29.93
22	72.36^ba^	30.58	27.64^ba^	30.58
23	64.21^b^	29.10	35.79^a^	29.10
24	67.23^ba^	31.09	32.77^ba^	31.09
25	67.00^ba^	30.53	33.00^ba^	30.53
Total	76.48	21.45	23.52	21.45

ab – means with different letters are significantly different at P-value ≤ 0.05

The analysis of variance showed significant differences in the degree of damage (determined using the comet assay) between individuals with normal DNA integrity. The lowest Head DNA% value was noted in cat No. 4 (98.29 ± 4.17). This cat differed significantly from most of the others in terms of damage shown by the Head DNA% result in the comet test. A level of damage comparable to that found in cat No. 4 was observed in seven individuals (1, 3, 5, 8, 18, 19 and 20). In the case of Tail DNA%, as could have been expected, the highest value was noted in cat No. 4 (1.71 ± 4.17), and it differed significantly from the results obtained in 17 cats. As in the case of Head DNA%, the DNA content in the tail of the comet in cat No. 4 was comparable to the level of damage in individuals 1, 3, 5, 8, 18, 19 and 20. The most stable genetic material in the group of cats with normal DNA integrity was found in individuals 15 and 24, with respective Head DNA% and Tail DNA% values of 99.98 ± 0.09 and 0.02 ± 0.09 in the former and 99.98 ± 0.08 and 0.02 ± 0.08 in the latter.

The statistical analysis showed significant differences in the degree of DNA damage between individuals with impaired DNA integrity. The highest values for Head DNA%, indicating the lowest degree of DNA fragmentation, were observed in six individuals (3, 4, 7, 10, 17 and 20), which did not differ significantly in this respect. Much greater damage (measured as Head DNA%) was observed in three individuals, Nos 1, 21 and 23, which had the lowest Head DNA%. The degree of damage in the remaining 16 cats was comparable and did not differ significantly from either the highest or lowest degree of damage. In three individuals (1, 21 and 23), the degree of damage measured as Tail DNA% was the highest and differed significantly from the level in individuals 3, 4, 7, 10, 17 and 20. In the remaining individuals, DNA damage measured as Tail DNA% was comparable.

Student’s *t*-test showed differences in the degree of DNA damage as determined by the comet assay (Table 7). The average degree of damage based on Head DNA% in cats with impaired DNA integrity (76.48%) was significantly higher than in cats with normal DNA integrity (99.62%), which was confirmed by Student’s *t*-test (*t* = 16.91 at P-value = 0.000). Student’s *t*-test (*t* = 17.01 at P-value = 0.000) also revealed significant differences between the average degree of DNA damage in cats with impaired DNA integrity (23.52%) and in cats with normal DNA integrity (0.37%) evaluated on the basis of Tail DNA%.

**Table 7.** Values of Student’s *t*-test of the differences between DNA in cats with impaired and normal DNA integrity evaluated using the comet assay

Health status	Head DNA (%)			Tail DNA (%)		
	Mean	*t*-test value	P-value	Mean	*t*-test value	P-value
Cats with normal DNA integrity	99.62	16.91	0.000	0.37	17.01	0.000
Cats with impaired DNA integrity	76.48			23.52		

## Discussion

Native electrophoresis of serum proteins divides this heterogeneous group into several basic fractions containing proteins with similar functions. In terms of diagnostics, the most important information obtained concerns disturbances in the concentrations of proteins in the α/β regions – containing mainly acute-phase proteins indicating ongoing inflammation – and in the γ region – containing primarily antibodies, *i.e*. indicating the existence of a humoral immune response. The results of the analysis should therefore be consistent with an identifiable cause of ongoing inflammation or source of antigen stimulation. In the present study, this connection was sought by considering the results in relation to a veterinarian’s assessment of the cat’s overall condition and to tests for infectious diseases. In all six cats with a positive plate-test result for one of the infectious diseases (FeLV, FIV or FIP), various deviations from references values were detected. In most of these cases, disturbances in various α-, β-, and γ-fractions were perceptible in native protein electrophoresis. One individual also had a low out-of-range concentration of the albumin fraction. It should be noted that individuals with overall condition assessed as good and which tested negative for infectious diseases also had protein fraction disturbances and unexplained changes in their blood counts.

In 2011, Gerou-Ferriani *et al*. (6) carried out serum protein gel electrophoresis in cats with lymphoma. These animals had lower albumin concentrations and higher β-globulin concentrations than healthy controls, although it was not shown that the sick animals always had elevated γ-globulin levels, and no characteristic electrophoretic patterns were observed. Samples taken from agarose gels were additionally identified using liquid chromatography–mass spectrometry, confirming the existence of the previously determined protein types in individual fractions.

Another study investigated cases of FIP infection in a large population of cats. In 65 cases of natural FIP infection, common clinicopathological changes included lymphopaenia (77%), neutrophilia (45%), anaemia (37%), hyperproteinaemia (39%) and hyperglobulinaemia (39%) (22). No differences were shown in the incidence of these abnormalities between 38 cases of effusive disease and 27 cases of non-effusive disease. The most consistent changes revealed by serum protein electrophoresis were increases in α_2_- and γ-globulin. The protein content in the exudates ranged from 39 to 98 g/L, with globulins ranging from 50% to 8%. Coronavirus serology showed wide variation in antibody titres (0 to 2,560), with 320 as the modal titre.

Hofmann-Lehmann *et al*. (8), in a long-term study, observed cats that were experimentally infected with FIV and/or FeLV and control cats for 6.5 years in identical conditions. Blood sample results of haematology, clinical chemistry and serum protein electrophoresis were compared. The haematological and clinical chemistry parameters changed significantly, especially in the FIV-positive animals, from month 9 after infection. The results of protein electrophoresis showed clear increases in the γ-globulin region.

Quantitative measurement of monoclonal serum protein (M protein) is one of the most important tools for monitoring disease activity in monoclonal gammopathies. In 2011, Mussap *et al*. (15) quantified M protein in serum using capillary zone electrophoresis and compared the results with those obtained by densitometric scanning in high-resolution agarose gel electrophoresis. The analysis was carried out using 82 samples taken from patients with various monoclonal gammopathies. All suspected M proteins were confirmed and characterised by immunofixation on an agarose gel.

A characteristic monoclonal peak is usually observed in the γ-globulin region, and sometimes in the β-globulin region as well. The result of this type of electrophoresis indicated the cause of problems in a cat with a rapidly growing subcutaneous mass (9). In this case, two characteristic peaks in the γ-globulin region were obtained. The types of antibodies – IgG and IgA – were confirmed by an immunofixation analysis. A reduced albumin concentration and a 0.23 albumin/globulin ratio (for a 1.0 reference limit) were observed as well. Subsequent histological analysis revealed that the condition was due to a plasma cell lymphoma infiltrating various tissues.

Seelig *et al*. (20) described the cases of two dogs with different symptoms: hypercalcaemia and acute anaemia. Determination of albumin and total globulin concentrations showed no deviations from reference ranges. However, electrophoretic analyses revealed the presence of monoclonal immunoglobulin A. On this basis, in conjunction with other clinicopathological results, IgA secretory neoplasms, B-cell lymphoma with plasmacytoid features and multiple myeloma were diagnosed. Despite the absence of marked disturbances of the protein fractions (albumins and total globulins) in basic tests, electrophoretic analysis was helpful in the diagnosis.

In the present study, single cell gel electrophoresis (the comet assay) was performed in 50 cats. Samples were taken from cats which showed deviations from reference standards in their blood counts, native protein electrophoresis, and plate tests for infectious diseases. Increased instability of genetic material was observed in all of these individuals. The amount of material present in the head of the comet (Head DNA%) ranged from 64.21% to 83.97%, depending on the individual. For comparison, 25 comet tests were performed in selected individuals from the group of 125 which did not show deviations from reference standards in the other tests. In this group, the percentage of stable DNA in the head of the comet ranged from 98.29 to 99.98. Thus a marked difference can be seen between the groups of animals. However, to the best of our knowledge, the comet assay is not used in veterinary laboratory diagnostics. It is used in environmental toxicological studies and in preclinical trials of medicinal products. It detects the level of instability of genetic material, and thus indirectly indicates the negative impact of various substances. To date it has not been used in veterinary laboratory diagnostics either in a panel of basic tests or as a supplementary test. The closest application is in semen quality analysis. Among tests of DNA integrity, the comet assay is a precise and sensitive test for detecting oxidative damage to genetic material (17). The aim of the study cited was to assess oxidative damage to sperm using the comet assay and to analyse the correlations with routine tests for assessing semen quality in dogs. A negative correlation was found between sperm motility and oxidative DNA damage. The authors conclude that given the high correlation with classical semen quality tests, the comet assay has high clinical and research potential in dogs.

An increase in DNA degradation is detected in many situations in which organisms are exposed to pollution. The alkaline version of the comet assay was used to estimate DNA integrity in blood samples and thus to determine whether DNA integrity in early life is associated with age, body weight, brood status and oxidative stress, using nestlings from a wild population of starlings (*Sturnus unicolor*) as a model organism (14). Significant levels of variation in DNA integrity were found, suggesting that it could affect the fitness of the offspring. The integrity of DNA depended on the stage of development; it was lower upon hatching than at the end of the nestling period. It was also negatively associated with the level of oxidative damage during hatching and positively associated with wing length at fledging. In addition, the birds positions in the size hierarchy at fledging matched differences in DNA integrity, with higher levels in core nestlings than in marginal ones. Finally, despite considerable variation in the individual with age, DNA integrity measurements in early life were shown to be moderately repeatable within broods. Therefore, DNA integrity in early life appears to be influenced mainly by environmental factors, such as natural stressors. The results suggest that measurement of variability in DNA integrity may be a useful approach to evaluating individual condition in natural populations and can be used in studies in developmental biology and ecology.

## Conclusion

Electrophoretic techniques are valuable analyses which provide extremely important information on an animal’s clinical condition. They are recommended in every case, including in screening tests of animals showing no concerning clinical symptoms. Screening by these means makes it possible to detect diseases displaying subclinical signals such as inflammation, antigen stimulation or some forms of cancer. Electrophoretic separation can be used successfully as an element of a panel of screening tests to assess the overall health condition of cats, and serum protein electrophoresis and single-cell electrophoresis emerge as useful diagnostic tools.

## References

[1] Burtis C.A., Ashwood E.R., Burtis C.A., Ashwood E.R. (2001). Tietz Fundamentals of Clinical Chemistry.

[2] Collins A.R. (2004). The comet assay for DNA damage and repair: principles, applications, and limitations. Mol Biotechnol.

[3] Comet Assay Software Project Laboratory CASPLab RRID:SCR_007249.

[4] Cray C., Zaias J., Altman N.H. (2009). Acute phase response in animals: a review. Comp Med.

[5] Errico G., Giordano A., Paltrinieri S. (2012). Diagnostic accuracy of electrophoretic analysis of native or defribrinated plasma using serum as a reference sample. Vet Clin Pathol.

[6] Gerou-Ferriani M., McBrearty A.R., Burchmore R.J., Jayawardena K.G.I., Eckersall P.D., Morris J.S. (2011). Agarose gel serum protein electrophoresis in cats with and without lymphoma and preliminary results of tandem mass fingerprinting analysis. Vet Clin Pathol.

[7] Giot J.F. (2010). Agarose gel electrophoresis – applications in clinical chemistry. J Med Biochem.

[8] Hofmann-Lehmann R., Cattori V., Tandon R., Boretti F.S., Meli M.L., Riond B., Lutz H. (2008). How molecular methods change our views of FeLV infection and vaccination. Vet Immunol Immunopathol.

[9] Igase M., Shimokawa Miyama T., Kambayashi S., Shimoyama Y., Hiraoka H., Hirata Y., Iwata M., Baba K., Mizuno T., Okuda M. (2016). Bimodal immunoglobulin A gammopathy in a cat with feline myeloma-related disorders. J Vet Med Sci.

[10] Knasmüller S., Steinkellner H., Hirschl A.M., Rabot S., Nobis E.C., Kassie F. (2001). Impact of bacteria in dairy products and of the intestinal microflora on the genotoxic and carcinogenic effects of heterocyclic aromatic amines. Mutat Res.

[11] Kotłowska M., Dietrich G., Wojtczak M., Karol H., Ciereczko A. (2007). Effects of liquid storage on amidase activity, DNA fragmentation and motility of turkey spermatozoa. Theriogenology.

[12] Kuchta-Gładysz M., Wójcik E.A., Słonina D., Grzesiakowska A., Otwinowska-Mindur A., Szeleszczuk O., Niedbała P. (2020). Determination of cytogenetic markers for biological monitoring in coypu (*Myocastor coypu*). Anim Sci J.

[13] Martinez-Subiela S., Tecles F., Montes A., Gutiérrez C., Cerón J.J. (2002). Effects of haemolysis, lipaemia, bilirubinaemia and fibrinogen on protein electropherogram of canine samples analysed by capillary zone electrophoresis. Vet J.

[14] Montoya B., Gil D., Valverde M., Rojas E., Pérez-Rodríguez L. (2020). DNA integrity estimated via the comet assay reflects oxidative stress and competitive disadvantage in developing birds. Physiol Biochem Zool.

[15] Mussap M., Pietrogrande F., Ponchia S., Stefani P.M., Sartori R., Plebani M. (2006). Measurement of serum monoclonal components: comparison between densitometry and capillary zone electrophoresis. Clin Chem Lab Med.

[16] Olive P.L., Banáth J.P. (2006). The comet assay: a method to measure DNA damage in individual cells. Nat Protoc.

[17] Pereira A.F., Borges P., Fontbonne A., Cardoso L., Gaivão I., Martins-Bessa A. (2017). The Comet assay for detection of DNA damage in canine sperm. Reprod Domest Anim.

[18] Pieper R., Gatlin C.L., Makusky A.J., Russo P.S., Schatz C.R., Miller S.S., Su Q., McGrath A.M., Estock M.A., Parmar P.P., Zhao M., Huang S.T., Zhou J., Wang F., Esquer-Blasco R., Anderson N.L., Taylor J., Steiner S. (2003). The human serum proteome: display of nearly 3700 chromatographically separated protein spots on two-dimensional electrophoresis gels and identification of 325 distinct proteins. Proteomics.

[19] Rossi S., Bertazzolo W., Paltrinieri S., Giordano A. (2008). Cellulose acetate electrophoresis of canine plasma after fibrinogen precipitation by ethanol. Vet Clin Pathol.

[20] Seelig D.M., Perry J.A., Avery A.C., Avery P.R. (2010). Monoclonal gammopathy without hyperglobulinemia in 2 dogs with IgA secretory neoplasms. Vet Clin Pathol.

[21] Singh N.P., McCoy M.T., Tice R.R., Schneider E.L. (1988). A simple technique for quantitation of low levels of damage in individual cells. Exp Cell Res.

[22] Sparkes A.H., Gruffydd-Jones T.J., Harbour D.A. (1991). Feline infectious peritonitis: a review of clinico-pathological changes in 65 cases, and a critical assessment of their diagnostic value. Vet Rec.

[23] Stockham S.L., Scott M.A. (2008). Fundamentals of Veterinary Clinical Pathology.

[24] Takiguchi M., Fujinaga T., Naiki M., Mizuno S., Otomo K. (1990). Isolation, characterization, and quantitative analysis of C-reactive protein from horses. Am J Vet Res.

[25] Tothova C., Nagy O., Kovac G. (2016). Serum proteins and their diagnostic utility in veterinary medicine: a review. Vet Med.

[26] Weiss D.J., Wardrop K.J., Weiss D.J., Wardrop K.J. (2010). Schalm’s veterinary hematology.

[27] Wójcik E.A., Kot E., Wójcik I., Wysokińska A., Matusevičius P. (2024). Genomic instability in the lymphocytes of dogs with squamous cell carcinoma. Animals.

[28] Wójcik E.A., Sokół A. (2020). Assessment of chromosome stability in boars. PLoS One.

[29] Wójcik E.A., Szostek M., Horoszewicz E., Kot E., Sałuch S., Smalec E. (2018). Analysis of chromatin instability of somatic cells in Sheep. Can J Anim Sci.

[30] Yamamoto S., Tagata K., Nagahata H., Ishikawa Y., Morimatsu M., Naiki M. (1992). Isolation of canine C-reactive protein and characterization of its properties. Vet Immunol Immunopathol.

